# CD47-CAR-T Cells Effectively Kill Target Cancer Cells and Block Pancreatic Tumor Growth

**DOI:** 10.3390/cancers9100139

**Published:** 2017-10-21

**Authors:** Vita Golubovskaya, Robert Berahovich, Hua Zhou, Shirley Xu, Hizkia Harto, Le Li, Cheng-Chi Chao, Mike Ming Mao, Lijun Wu

**Affiliations:** 1 Promab Biotechnologies, Richmond, CA 94806, USA; Robert.berahovich@promab.com (R.B.); huazhou369@gmail.com (H.Z.); shirley.xu@promab.com (S.X.); hizkia.harto@promab.com (H.H.); simon.li@promab.com (L.L.); john@promab.com (L.W.); 2 Forevertek Biotechnology, Changsha 410003, China; 3 GenoImmune/a BGI’s company, Shenzhen 518083, China, cchichao@genomics.cn; 4 Antagene Inc., Santa Clara, CA 95054, USA; mikemao@antagene.com

**Keywords:** chimeric antigen receptor (CAR), cell therapy, immunotherapy, CD47 tumor antigen, humanized antibody

## Abstract

CD47 is a glycoprotein of the immunoglobulin superfamily that is often overexpressed in different types of hematological and solid cancer tumors and plays important role in blocking phagocytosis, increased tumor survival, metastasis and angiogenesis. In the present report, we designed CAR (chimeric antigen receptor)-T cells that bind CD47 antigen. We used ScFv (single chain variable fragment) from mouse CD47 antibody to generate CD47-CAR-T cells for targeting different cancer cell lines. CD47-CAR-T cells effectively killed ovarian, pancreatic and other cancer cells and produced high level of cytokines that correlated with expression of CD47 antigen. In addition, CD47-CAR-T cells significantly blocked BxPC3 pancreatic xenograft tumor growth after intratumoral injection into NSG mice. Moreover, we humanized mouse CD47 ScFv and showed that it effectively bound CD47 antigen. The humanized CD47-CAR-T cells also specifically killed ovarian, pancreatic, and cervical cancer cell lines and produced IL-2 that correlated with expression of CD47. Thus, CD47-CAR-T cells can be used as a novel cellular therapeutic agent for treating different types of cancer.

## 1. Introduction

Immunotherapy using engineered T cells modified with chimeric antigen receptor (CAR) has been shown to be a promising therapy against hematological cancers and also solid tumors [1,2,3,4]. CAR consists of an extracellular antigen-binding domain such as single-chain antibody variable fragment, called ScFv, fused to a hinge, transmembrane domain, co-stimulatory domains (CD28, 4-1BB, CD27 or other), and CD3 ζ activation domains [5]. Recently several clinical trials showed highly promising results using CAR-T cells against different types of cancer [6,7,8]. The clinical trials with CD19-CAR-T cells targeting CD19 antigen demonstrated excellent results in patients with B cell acute lymphoblastic leukemia (B-ALL), with an overall response rate of 88% [6]. There are many clinical trials with CAR-T cells targeting not only hematological cancer targets (such as CD19, CD22, BCMA) but also solid tumor targets (such as EGFR, EGFRvIII, mesothelin, Her-2, CEA, PSMA and others) [3]. The different approaches and the discovery of new antigens are important to improve CAR-T cell therapy targeting solid tumors [9].

CD47 is a cell surface glycoprotein of the immunoglobulin superfamily that is often overexpressed in both hematological cancers (leukemia, lymphoma [10], and multiple myeloma) and solid cancers such as ovarian, small cell lung cancer, pancreatic, glioma, glioblastoma, pediatric brain tumors and other types of cancers [10,11]. CD47 is also known as a “don’t eat me signal” through binding to SIRP-α (signaling regulatory protein alpha) and blocking phagocytosis of tumor cells mediated by SIRP [12,13]. High expression of CD47 has been shown to correlate with poor clinical outcomes in patients with hematological cancers such as non-Hodgkin lymphoma [10] or acute myeloid leukemia (AML) [14] and also in solid tumors (ovarian, glioblastoma, glioma, and others) [15] and proposed to be a clinical prognostic factor. In addition, CD47 signaling has been shown to play a key role in maintenance of tumor initiating or cancer stem cells [16].

The CD47 pathway was successfully blocked with mouse antibodies [15], humanized antibodies [17] or completely human antibodies [18] against CD47 antigen that resulted in blocking tumor growth using cancer cell line and patient-derived xenograft mouse models [11]. Another anti-cancer therapeutic approach to block CD47 was demonstrated with the use of CD47 siRNA [19] or CD47 shRNA [17]. The authors demonstrated that intravenous delivery of CD47 siRNA blocked melanoma tumor growth and lung metastases [19]. Inhibition of CD47 with shRNA induced the presence of macrophages [17]. Inhibition of CD47 signaling has been shown to induce SIRP-alpha-dependent phagocytosis [14] and also other SIRP-alpha-independent pathways such as interaction with the EGFR pathway or TSP-1/VEGFR-2 pathway [16,20,21]. Importantly, inhibition of the CD47 pathway with CD47 antibodies or other agents preferentially targeted tumor cells of both hematological and solid tumors rather than normal cells [14,17] suggesting that there is a “therapeutic window” for CD47-targeting therapeutics and that there are differences between cancer and normal cells in CD47-dependent signaling. Thus, CD47 targeting is a highly promising therapeutic approach.

In this report, we demonstrate the efficacy of CD47-CAR-T cells against several cancer cells: ovarian, lung, hepatocellular carcinoma and pancreatic cancer cell lines. The CD47-CAR-T cells demonstrated cytotoxic activity against cancer cell lines and produced cytokines that correlated with the level of CD47 expression. In addition, intratumoral injection of CD47-CAR-T cells significantly decreased BxPC3 xenograft tumor growth using NSG mice. In addition, we developed humanized CD47 ScFv based on the mouse CD47 ScFv VH and VL sequences and found that humanized CD47 ScFv bound CD47 human extracellular antigen. We engineered humanized CD47-CAR-T cells and demonstrated that it also effectively and specifically killed CD47-positive cancer cells. Thus, CD47 and humanized CD47-CAR-T cells can be used as a novel immunotherapy approach to eliminate CD47-positive cancers.

## 2. Results

### 2.1. CD47 Mouse Antibody Detects CD47 Human Antigen that Has Variable Expression in Different Cancer Cell Lines

**Figure 1:**
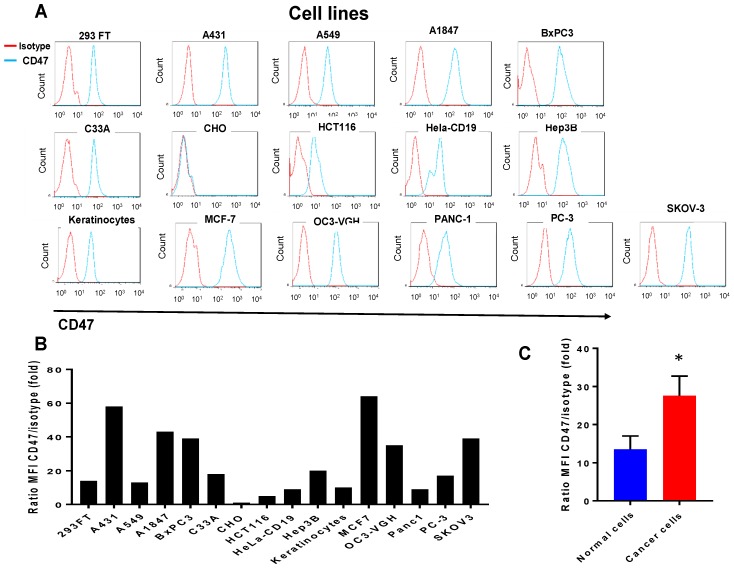
Variable expression of CD47 in different cell lines. (**A**) The FACS staining was done with CD47 (B6H12) (BioLegend, San Diego, CA, USA) antibody at 10 μg/mL as described in Materials and Methods. Different cancer cell lines, as well and normal cell lines—keratinocytes, 293FT and CHO cell lines—were analyzed. The representative FACS plots from one to six independent experiments are shown; (**B**) Quantification of MFI to isotype control is shown. The MFI quantitation of data presented on Figure 1A show ratio of CD47 staining to isotype control; (**C**) Cancer cell lines express significantly higher levels of CD47 than normal cells. The bars show average ratio MFI CD47 staining to isotype control in human cancer cell lines and normal cell lines +/− standard errors from above experiments. * *p* = 0.023, Student’s *t*-test. Please define.

We used mouse monoclonal B6H12 CD47 antibody to detect CD47 in different cancer cells line by FASC staining (Figure 1A). CD47 antibody detected a higher level of CD47 expression in cancer cell lines versus normal keratinocytes and 293 cells (Figure 1A–C). It did not recognize expression in CHO cells, was low in cervical Hela-CD19 cells (Hela cells with stable CD19 expression) and colon cancer HCT116 cells as well as normal keratinocytes. The expression was moderate in lung cancer A549; cervical cancer, C33A; prostate cancer, PC-3 and high in squamous cell carcinoma, A431; breast cancer, MCF-7; ovarian cancer SKOV-3 cell line and pancreatic cancer cell line BxPC3 cells. Thus, CD47 antibody effectively detected CD47 antigen demonstrating variable CD47 expression in different cancer cell lines that was in most cases higher than in non-cancerous cell lines such as keratinocytes and 293 cells (Figure 1A,B). The average expression of CD47 in all tested cancer cells lines was significantly higher than in normal cell lines (Figure 1C).

### 2.2. Mouse CD47 ScFv Effectively Binds CD47 Antigen and CD47-CAR-T Cells Kill CD47-Positive Cancer Cells

**Figure 2:**
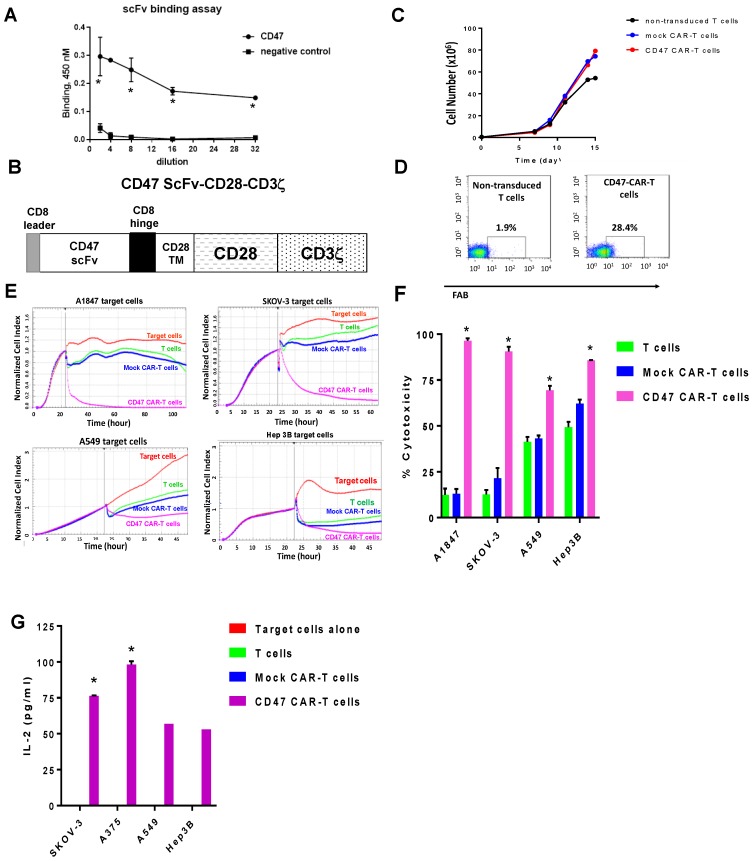
CD47 ScFv from mouse antibody binds CD47 and CD47-CAR-T cells effectively kill cancer cells in a CD47-dependent manner and produce IL-2. (**A**) ELISA binding analysis of CD47ScFv and CD47 antigen. Negative control CD49 antigen was used. There is significantly higher dose-dependent binding of CD47ScFv (B6H12) with CD47 antigen compared with negative control. * *p* < 0.05, Student’s *t*-test. Data are shown from two independent experiments; (**B**) The structure of CD47-CAR. The second generation CAR with CD28 co-stimulatory domain was used; (**C**) CD47 expansion in vitro. CD47-CAR-T cells expanded for >14 days similarly to control non-transduced T cells and Mock-CAR-T cells. Representative growth curve from two independent experiment is shown; (**D**) Expression of CD47-CAR in T cells transduced with lentiviral CD47-CAR by FACS with F(ab)2 antibody. T cells were effectively transduced with CD47-CAR. Expression of CAR is confirmed by FACS with F(ab)2 antibody and compared with negative control non-transduced T cells. Representative FACS data are shown from two independent experiments; (**E**) The Real-time cytotoxicity assay (RTCA) demonstrates that CD47-CAR-T cells kill CD47-positive cancer cells more significantly than CD47-low expression cancer cells; (**F**) Cytotoxicity of CD47-CAR-T cells versus T cells and Mock-CAR-T cells. The quantitation of RTCA from three independent experiments is shown. * *p* < 0.0001 CD47-CAR-T cells versus T cells and Mock CAR-T cells by 2-way ANOVA with Tukey’s post-test. G. CD47-CAR-T cells produce IL-2 in a CD47-dependent manner; high in CD47-positive cells and lower in CD47-negative cells. The Effector to target E:T ratio was 1:1. The bars show average IL-2 secretion by CD47 CAR-T cells from two independent experiments. * *p* < 0.05, Student’s *t*-test in SKOV-3 and A375 cells versus T cells, Mock CAR-T cells, A549 cells and Hep 3B cells.

We tested ScFv from mouse monoclonal antibody B6H12 for binding activity with human CD47 antigen. Mouse CD47scFv bound human CD47 antigen, while it did not bind negative control, CD49 antigen (Figure 2A).

The CD47 ScFv was used to generate second generation CAR-T cells, CD47-CAR-T cells with CD28 co-stimulatory domain and CD zeta activation domain (Figure 2B). The CD47-CAR-T cells expanded >150-fold on day 14 in vitro (Figure 2C) and expressed CAR as demonstrated by FACS with F(ab)2 antibody (Figure 2D). To test the killing activity in vitro, we tested CD47 CAR-T cells by RTCA assay (Real-time cytotoxicity assay) with CD47-positive cells expressing a high level of CD47 antigen: ovarian cancer cells, A1847, and SKOV-3 and with cancer cells expressing a low level of CD47: A549 cells and Hep3B cells (Figure 2E). The CD47-CAR-T cells effectively killed high-expressing cancer cell lines such as A1847, SKOV-3, and CAR-T cells killed much less A549 and Hep3B and cells with a lower level of CD47 versus T cells or Mock-CAR-T cells (Figure 2E). The cytotoxicity of cancer cells was significantly higher than T cells and Mock-CAR-T cells, *p* < 0.0001 (Figure 2F). This demonstrates CD47-dependent activity of CD47-CAR-T cells depending on expression of CD47 antigen.

The CD47-CAR-T cells produced Il-2 cytokine against cancer cells that was significantly higher in SKOV3 cells, highly positive for CD47 than in A549 and Hep3B cells with lower expression of CD47 (Figure 2G). Thus, CD-47-CAR-T cells kill and secrete IL-2 cytokine in a CD47-dependent manner based on CD47 expression on the surface of cancer cells that is consistent with cytotoxicity data.

### 2.3. CD47-CAR-T Cells Significantly Decrease BxPC3 Pancreatic Cancer Xenograft Tumor Growth

**Figure 3:**
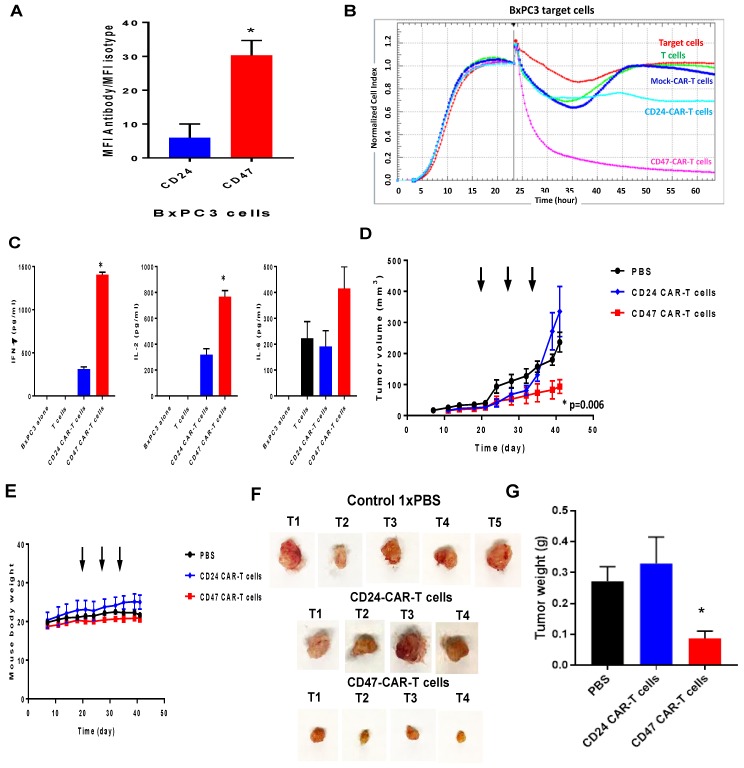
CD47-CAR-T cells significantly decrease BxPC3 pancreatic cancer xenograft tumor growth. (**A**) CD47 expression is significantly higher than CD24 expression in BxPC3 pancreatic cancer cells. The bars show average ratio of MFI to isotype control IgG1 of CD24 and CD47 expression in BxPC3 cells ± standard errors from two independent experiments. * *p* = 0.029 CD47 versus CD24, Student’s *t*-test; (**B**) CD47-CAR-T cells effectively killed BxPC3 cancer cell lines by RTCA assay. The control CD24-CAR-T cells and Mock Control CAR-T cells did not significantly kill BxPC3 cancer cells in three independent experiments. The CD47-CAR-T cell cytotoxicity was significantly higher than control cells in BxPC3 cells, *p* < 0.05, Student’s *t*-test; (**C**) CD47-CAR-T cells secreted high levels of cytokines: IFN-gamma, IL-2 and IL-6 against BxPC3 cells in vitro that was consistent with high cytotoxic activity of CD47-CAR-T cells against BxPCR3 cells shown in Figure 3B. The supernatants from RTCA assay were used for cytokine ILISA assay. E:T=10:1. The IL-2 and IFN-gamma cytokine levels were significantly higher in CD47-CAR-T cell samples versus control CD24-CAR-T cells, *p* < 0.05, Student’s *t*-test; (**D**) CD47-CAR-T cells significantly decreased BxPC3 tumor growth, * *p* = 0.006, CD47-CAR-T cells versus 1xPBS control. *n* = 4–5 mice, CD47/CD24-CAR-T cells and 1xPBS groups, respectively; (**E**) CAR-T cells did not affect mice weight in CD47-CAR-T cell, CD24-CAR-T cell and 1xPBS control groups. Mice weight was measured in grams two times a week; (**F**,**G**) CD47 CAR-T cells significantly decreased tumor size and weight, respectively. *p* < 0.05, CD47-CAR-T cells versus control CD24-CAR-T cells and 1xPBS groups, Student’s *t*-test. At the end of the experiment mouse tumors were analyzed by imaging (**F**) and measuring weight in grams (**G**).

To test in vivo efficacy of CD47-CAR-T cells, we used BxPC3 pancreatic cancer cells. We compared CD47-CAR-T cytotoxicity with Mock CAR-T control cells and CD24-CAR-T cells. CD24-CAR-T cells with CD24-CAR ScFv were used as non-CD47 control CAR-T cells based on significantly lower expression of CD24 in BxPC3 cells compared to CD47 (Figure 3A). The CD47-CAR-T cells expressed high cytotoxic activity against BxPC3 cells compared with Mock control CAR-T cells and CD24-CAR-T cells (Figure 3B).

In addition, CD47-CAR-T cells expressed a significantly higher level of cytokines: IFN-gamma, IL-2 versus Mock and CD24-CAR-T cells (Figure 3C), and expressed a high level of IL-6 against target BxPC3 cells versus Mock Control and CD24-CAR-T cells (Figure 3C). We injected BxPC3 cancer cells subcutaneously into NSG mice to generate established xenograft tumors (Figure 3D). Then three injections of CD47-CAR-T cells, control 1× PBS and CD24-CAR-T cells were applied intra-tumorally at days 20, 27 and 34. CD47-CAR-T cells significantly decreased BxPC3 xenograft tumor growth compared with controls, *p* < 0.05 (Figure 3D). CD47-CAR-T cells did not affect mice weight (Figure 3E). The tumor size (Figure 3F) and weight (Figure 3G) from the CD47-CAR-T cell-treated group were significantly less (*p* < 0.05) than from the control 1× PBS and CD24-CAR-T cell groups.

**Figure 4:**
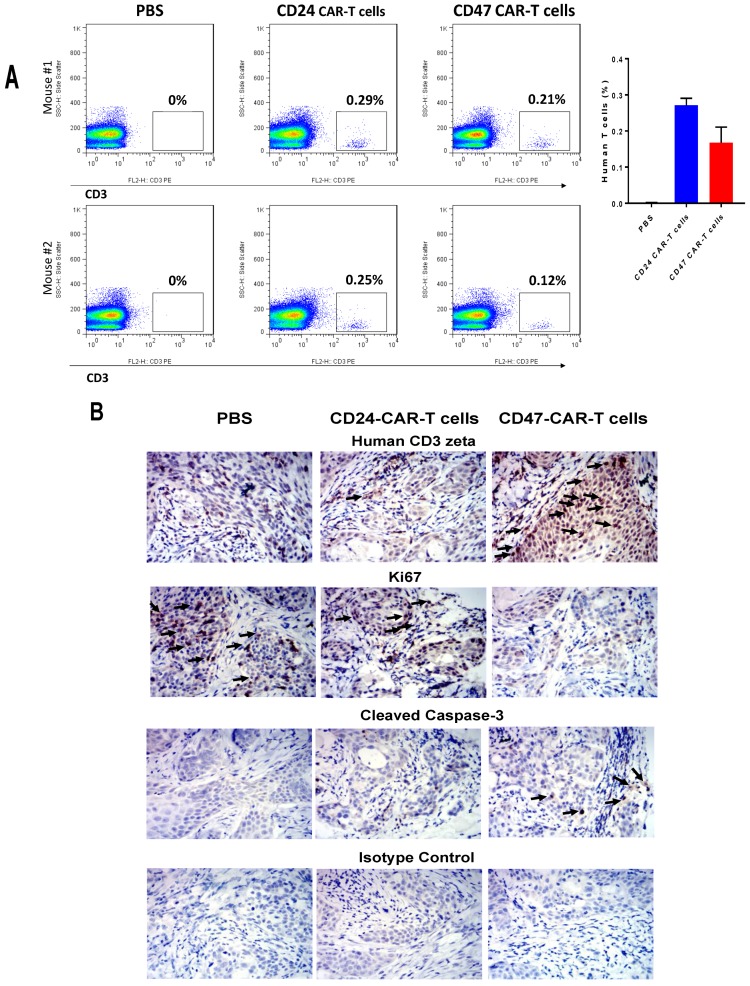
FACS staining of mouse blood cells and IHC of tumor samples detects presence of human T cells in blood and increase in tumors, decreased level of Ki67 and increased level of caspase-3. (**A**) FACS staining of mouse blood cells demonstrates significantly increased level of human T cells in CD47-CAR-T and CD24-CAR-T cells groups among all T cells. * *p* < 0.03; (**B**) IHC staining with CD3 zeta antibody demonstrates increased staining in CD47-CAR-T samples versus control 1xPBS and CD24-CAR-T cells (upper panels); IHC staining with Ki67 antibody demonstrates decreased Ki67 level in CD47-CAR-T samples versus control 1xPBS and CD24-CAR-T cells samples. IHC with caspase-3 antibody demonstrates increased level of cleaved caspase-3 in CD47-CAR-T cells treated tumors versus control tumor samples; The IHC staining with isotype control IgG1 antibody was negative in three groups (lower panels). Black arrows show differences in IHC staining.

The blood of mice treated with CD47-CAR-T cells and CD24-CAR-T cells detects presence of human T cells in mice blood (Figure 4A). The level of human T cells was low (<0.2%) for CD47-CAR-T cells among all mice T cells. To test the level of human T cells inside mice xenograft tumors we used IHC staining of xenograft tumors with human CD3 zeta antibody. The CD3 zeta staining was higher in CD47-CAR-T-treated mice versus control 1× PBS-treated and CD24-CAR-T-treated group (Figure 4B, upper panels, marked by arrows), while proliferation marker Ki67 staining was lower in CD47-CAR-T tumors versus control groups (Figure 4B).

In addition, the level of cleaved caspase-3 was significantly increased in CD47-CAR-T cells versus control groups (Figure 4B). The negative control isotype was negative in both CD47-CAR-T cell-treated and control 1× PBS and CD24-CAR-T cell-treated mice samples (Figure 4B).

### 2.4. Humanized CD47 ScFv Effectively Binds CD47 Antigen and Detects CD47 in Tumor Samples

**Figure 5:**
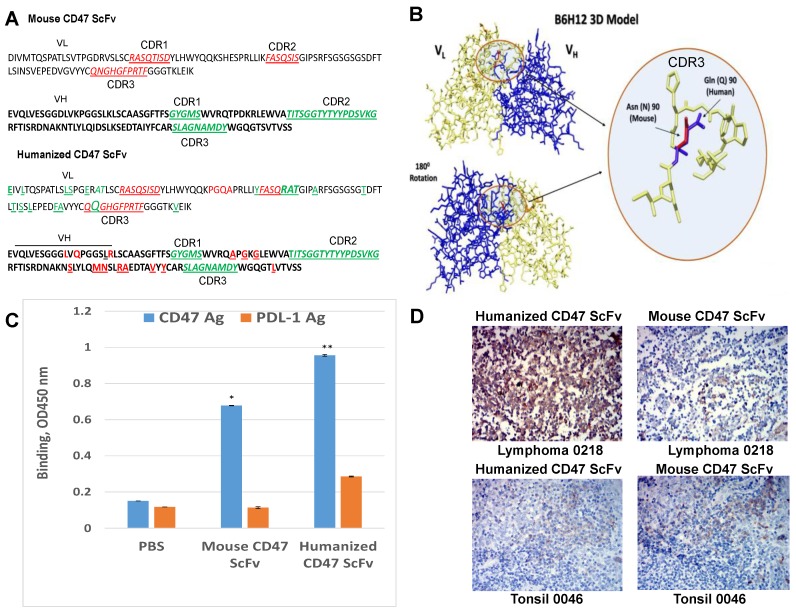
Humanized CD47 VL and VH sequences, binding of humanized CD47 ScFv with CD47 antigen and IHC staining of human tumor and normal tissues. (**A**) Amino-acid sequence of humanized CD47 ScFv. Upper panel. Mouse CD47 ScFv of B6H12 antibody. CDR 1,2, and 3 regions of VL underlined and marked by red. CDR 1,2, and 3 regions of VH underlined and marked by green. Lower panel: Humanized CD47 is shown. The differences between humanized and mouse sequences in frame regions and CDR regions are shown by red or green; (**B**) Computer in silico model of humanized VL and VH regions. In circle differences in CDR3 region of VL between humanized and mouse sequence is shown; (**C)** ELISA demonstrates high binding activity of humanized CD47 ScFv with CD47 antigen. There was no high activity with negative control human PDL-1 antigen. The binding activity was significantly higher than mouse CD47 ScFv. The bars show average of binding activity from two independent experiments. * *p* = 0.0025, mouse CD47 versus PDL-1 control; ** *p* = 0.0025 humanized CD47 vs. PDL-1 control; and *p* = 0.0028 humanized CD47 vs. mouse CD47 by Student’s *t*-test analysis; (**D**) IHC staining with humanized CD47 ScFv and mouse CD47 ScFv of tumor and normal samples. Similar staining is observed. IHC with humanized CD47 ScFv demonstrates high staining in tumor samples and low staining in normal tissues.

The bioinformatics approach was performed to humanize VH and VL of the CD47 antibody B6H12 described by [22,23]. Four different VH and VL were used for design of the humanized CD47 ScFv and testing binding with human CD47 antigen. The humanized version of CD 47 ScFv with a high affinity to the CD47 antigen is shown in Figure 5A; it had the same CDR 1, 2, 3 regions of heavy chain, VH as the mouse B6H12 antibody (CDRs regions are shown in green color) and human frame regions similar to mouse antibody, with differences shown in red. The humanized version of CD47 antibody had three amino-acid changes in CDR2 and one amino acid changes in CDR3 region in VL versus mouse B6H12 (CDRs are shown in red and changes in CDR are shown in green) (Figure 5A). The humanized CD47 antibody model is shown in Figure 5A with the change of glutamine, Q instead of asparagine, N as in mouse antibody shown in the circle (Figure 5B). We tested binding of humanized CD47 ScFv by ELISA (Figure 5C). It had significantly higher binding than mouse CD47 ScFv and had higher binding than negative control PDL-1 (Figure 5C). The humanized CD47 ScFv detected CD47 antigen better in lymphoma samples and similarly in other tumor samples (ovarian, gastric cancer (not shown) and less or similarly in normal (Figure 5D).

### 2.5. Humanized CD47-CAR-T Cells Effectively Kill Cancer Cells in a cd47dependent Manner

**Figure 6:**
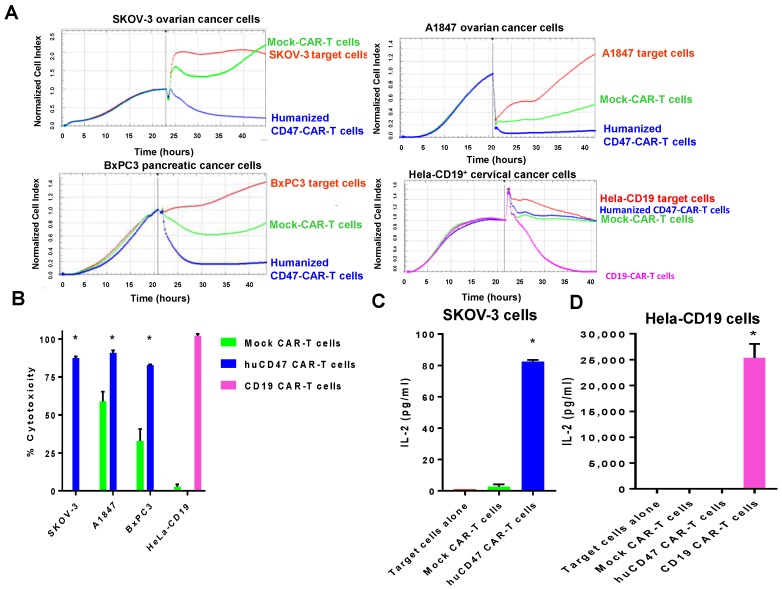
Humanized CD47 CAR-T cells are highly cytotoxic against CD47-positive cancer cells. (**A**) RTCA cytotoxicity assay with humanized CD47-CAR-T cells and CD47-positive or CD47-negative Hela-CD19 cancer target cells. CD47-CAR-T cells cytotoxic against CD47-positive but not against CD47-negative Hela-CD19 cells. Positive control CD19-CAR-T cells effectively kill CD19-positive Hela-CD19 cells; (**B**) Quantification of RTCA assay show significantly increased CD47-CAR-T cell cytotoxicity with CD47-positive cancer cell lines but not with CD47-negative Hela-CD19 cells. * *p* < 0.001, CD47-CAR-T cells versus Mock-CAR-T cells, Student’s *t* test. Bars show average percent of cytotoxicity ± standard deviations in three independent experiments; (**C**) Increased secretion of IL-2 by humanized CD47-CAR-T cells with CD47-positive cells SKOV-3 cells. E:T ratio=1:1. *p* < 0.05, CD47-CAR-T cells versus controls, Student’s *t*-test, *n* = 3; (**D**) IL-2 is not secreted by CD47-CAR-T cells with CD47-hegative cells, Hela-CD19 cells. Mock-CAR-T cells are negative control cells. CD19-CAR-T cells are positive control cells against Hela-CD19 target cells. E:T ratio=10:1. *p* < 0.05, CD19-CAR-T cells versus controls, Student’s *t*-test, *n* = 3.

We designed humanized CD47-CAR construct the same way as shown in Figure 2B and tested these cells against CD47 high-expressing ovarian: SKOV-3, A1847 and pancreatic, BxPC3 cancer cell lines, and low-expressing Hela-CD19 cancer cell line (Figure 6A). Humanized CD47-CAR-T cells effectively killed CD47-positive cancer cells and had no killing activity against Hela-CD19 cancer cells with very low expression of CD47, while positive control CD19-CAR-T cells effectively killed Hela-CD19 cells (Figure 6A, lower right panel). The CD47-CAR-T cytotoxicity was significantly increased in CD47-positive cells versus CD47-negative cells and control Mock CAR-T cells (*p* < 0.05) (Figure 6B). The IL-2 cytokine level secreted by CD47-CAR-T cells was also increased significantly (*p* < 0.05) in SKOV-3-CD47-positive cell line versus Mock-CAR-T cells (Figure 6C), but was not present in Hela-CD19 cells, negative for CD47 (Figure 6D). In contrast, control CD19-CAR-T cells produced significantly (*p* < 0.05) increased level of IL-2 in Hela-CD19-positive cells versus Mock-CAR-T cells (Figure 6D). Thus, humanized CD47-CAR-T cells effectively and specifically killed cancer cell lines and produced cytokines in a CD47-dependent manner.

## 3. Discussion

The present report demonstrates a high activity of CD47-CAR-T cells against cancer cell lines with a high expression of CD47. In addition, it demonstrates that intra-tumor injection of CD47-CAR-T cells significantly decreased pancreatic BxPC3 xenograft tumor growth versus two controls: 1xPBS and CD24-CAR-T cells that were accompanied by increased human CD3 zeta IHC staining in CD47-CAR-T cell treated tumors versus control tumors. Moreover, Ki67 was decreased and caspase-3 was activated in CD47-treated xenograft tumors. We also designed humanized CD47 ScFv and showed its binding to human CD47 antigen by both ELISA and IHC staining using human tumor samples. The humanized CD47-CAR-T cells effectively killed cancer cell lines with a high expression of CD47 and did not kill and did not produce cytokines in CD47-negative cancer cells. Thus, CD47-CAR-T cells and humanized CD47-CAR-T cells can be a novel type of cellular therapy for solid and hematological cancers.

CD47 is a cell surface antigen that is often overexpressed in tumor samples and plays multiple roles in tumor growth, metastasis and immunoregulation [24]. It plays a critical role in blocking phagocytosis of CD47-positive tumors and produced cytokines [15]. Blocking interaction of CD47 and SIRP alpha on macrophages with CD47 mouse [25], humanized or human antibodies [18] induced phagocytosis of CD47-positive tumors. There were some reports on CD47 siRNA [19] that down-regulated CD47 and blocked tumor growth and also other reports demonstrated CD47-SIRP-alpha-independent pathways that blocked tumorigenesis such as interaction with thrombospondin, TSP-1, VEGFR-2 or EGFR [26]. This is the first report, demonstrating the efficacy of CD47-CAR-T cells against solid tumors.

This report shows a high specificity of CD47-CAR-T cells to CD47-positive cancer cells with a high expression of CD47 and an absence of CAR-T activity in target cells with low expression of CD47 such as Hela-CD19 cells. Thus, a therapeutic window exists where CD47-CAR-T cells affect only highly expressing CD-47 cells. In addition, CD47-CAR-T cells did not decrease mouse body weight, suggesting no toxic effect from CAR-T cells, although more detailed toxicology studies will be required to increase the safety of this therapy. We would like to note that CD47 also is expressed in normal cells and normal tissues as we detected by FACS and IHC staining. Thus, different approaches of CAR-T cell regulation such as switch-on, switch-off mechanisms; bi-specific CARs; regulation of cytokines involved in cytokine release syndrome and other approaches should be applied to increase safety and to overcome the major challenges of this CAR-T cell therapy. Intra-tumor injection of CD47-CAR-T cells increases the safety of these cells. We did detect an increased level of human CD47-CAR-T cells in mouse blood in CD47-CAR-T cell treated mice, but their percentage was low <0.2% (Figure 4). The majority of CD47-CAR-T cells were localized inside tumors (Figure 5). Thus, regional intra-tumor delivery of CD47-CAR-T cells is a feasible approach in clinic. In fact, recent studies demonstrated several clinical trials with intra-tumor delivery of CAR-T cell [27,28]. The authors demonstrated the superior efficacy of regional IP (intraperitoneal) infusion of CEA-CAR-T cells versus systemically infused CAR-T cells [28]. The regional delivery of CAR-T cells can be done in many types of tumors: glioblastoma [29], hepatic metastases of colorectal carcinoma [30], head and neck, and pleural mesothelioma [31]. Thus, regional delivery of CD47-CAR-T cells and others can be advantageous for increased safety, direct delivery of CAR-T to tumors with less repressive effects of microenvironments and other mechanisms [27].

CD47 is also highly expressed in cancer stem cells, the most aggressive type of tumor cells [32]. Thus, CD47-CAR-T cells can be used to target cancer stem cells. In fact we showed that ovarian SKOV-3 cancer spheres express a high level of CD47 tumor antigen, and future study will elucidate the mechanisms of CD47-ACR-T cells against CD47-positive cancer stem cells.

Thus, this is the first report demonstrating a novel approach to target cancer cells with CD47-CAR-T cells. It demonstrates the high efficacy of CD47-CAR-T cells against cancer cells in vitro and *in vivo* and provides a novel anti-cancer cellular therapeutics.

## 4. Materials and Methods

### 4.1. Cell Lines

Hela cells were purchased from the ATCC (Manassas, VA, USA) and cultured in DMEM with 10% FBS and 1% penicillin/streptomycin. Hela-CD19 cells with stable expression of CD19 were maintained in DMEM with 10% FBS, puromycin and penicillin/streptomycin.as described in [33]. Human peripheral blood mononuclear cells (PBMC) were isolated from whole blood obtained in the Stanford Hospital Blood Center (Stanford, CA, USA) according to IRB-approved protocol using Ficoll-Paque solution (GE Healthcare, Chicago, IL, USA). HEK293FT cells from AlStem (Richmond, CA, USA) were cultured in DMEM containing 10% FBS and penicillin/streptomycin. SKOV-3 cell line was obtained from ATCC and cultured in RPMI plus 10% FBS and penicillin/streptomycin. Normal human keratinocytes were obtained from the Lonza company and cultured in keratinocyte medium (Lonza, Anaheim, CA, USA) according to the manufacturer’s protocol. BxPC3, PANC-1, A1847, A375, A549 and Hep-3 B were obtained from ATCC and cultured in DMEM with 10% FBS and penicillin/streptomycin. The cell lines were authenticated by flow cytometry in our laboratory, using cell-specific surface markers or by ATCC.

### 4.2. CAR Constructs

CD47 ScFv from B6H12 antibody was inserted using Nhe I and Xho I s between CD8-alpha signaling peptide, and CD8-alpha hinge, with down-stream fused CD28 transmembrane domain, CD28 co-activation domain and CD3 activation domain. This CD47-CAR construct was flanked by Xba I and Eco R I sites in pCD510 lentiviral vector (Systems Bioscience, Palo Alto, CA, USA). The same construct was done with humanized CD47 ScFv and Mock control ScFv of intracellular protein, called humanized CD47 and Mock-CAR, respectively. CD24 ScFv from Promab Biotechnologies (Richmond, CA, USA) CD24 (clone 4F4E10) antibody was used as above for the CD24-CAR construct. The CAR were synthesized and sequenced in both directions by Syno Biological (Beijing, China).

### 4.3. CAR Lentivirus Preparation

The lentiviral CARs were used for generation of lentivirus using 293 FT cells, Lentivirus Packaging Mix and transfection agent (Alstem, Richmond, CA, USA) as described [33]. The virus titers were determined by quantitative RT-PCR using the Lenti-X qRT-PCR kit (Takara Bio, Mountain View, CA, USA) according to the manufacturer’s protocol and the 7900HT thermal cycler (Thermo Fisher Scientific, South San Francisco, CA, USA). The lentiviral titers were expressed in pfu/mL and ranged 1–10 × 10^8^ pfu/mL.

### 4.4. CAR-T Cells Expansion

PBMC were suspended at 1 × 10^6^ cells/mL in AIM V-AlbuMAX medium (Thermo Fisher) containing 10% FBS with 300 U/mL IL-2 (Thermo Fisher). PBMC were activated with an equal number of CD3/CD28 Dynabeads (Thermo Fisher), and cultured in non-treated 24-well plates. At 24 and 48 h, lentivirus was added to the cultures at a multiplicity of infection (MOI) of 5 with 1 μL of TransPlus transduction enhancer (AlStem). The CAR-T cells were counted every 2–3 days and fresh medium with 300 U/mL IL-2 was added to the cultures to maintain the cell density at 1 × 10^6^ cells/mL.

### 4.5. Flow Cytometry

To measure CAR expression, 5 × 10^5^ cells were suspended in 100 μL of buffer (1× PBS with 0.5% BSA) and incubated on ice with 1 μL of human serum (Jackson Immunoresearch, West Grove, PA, USA) for 10 min. Then 1 μL of allophycocyanin (APC)-labeled anti-CD3 (eBioscience, San Diego, CA, USA), 2 μL of 7-aminoactinomycin D (7-AAD, BioLegend, San Diego, CA, USA), and 2 μL of anti-F(ab)2 or its isotype control was added, and the cells were incubated on ice for 30 min. The cells were rinsed with buffer, and acquired on a FACSCalibur (BD Biosciences, San Jose, CA, USA). Cells were analyzed first for light scatter versus 7-AAD staining, then the 7-AAD^−^ live gated cells were plotted for CD3 staining versus F(ab)2 staining or isotype control staining. In some experiments anti-F(ab)2 staining alone was done. For the mouse tumor studies, 100 μL of blood was stained at room temperature for 30 min with 1 μL of APC anti-CD3, 2 μL of fluorescein isothiocyanate (FITC)-labeled anti-CD8a (eBioscience), 2 μL of 7-AAD. Erythrocytes were lysed with 3.5 mL of RBC lysing solution (150 mM NH_4_Cl, 10 mM NaHCO_3_, 1 mM EDTA pH 8), then leukocytes were collected by centrifugation and rinsed with 2 mL of cold buffer before acquisition. For expression of CD47, mouse B6H12 antibody from (BioLegend) that was used according to the manufacturer’s protocol at 10 μg/mL concentration. For expression of CD24, mouse CD24 clone 4F4E10 (Promab Biotechnologies, Richmond, CA, USA) was used at 10 μg/mL. The isotype IgG1 isotype antibody was used at 10 μg/mL from (BioLegend).

### 4.6. Real-Time Cytotoxicity Assay (RTCA)

Adherent target cells were seeded into 96-well E-plates (Acea Biosciences, San Diego, CA, USA) at 1 × 10^4^ cells per well and monitored in culture overnight with the impedance-based real-time cell analysis (RTCA) iCELLigence system (Acea Biosciences). The next day, the medium was removed and replaced with AIM V-AlbuMAX medium containing 10% FBS ± 1 × 10^5^ effector cells (CAR-T cells or non-transduced T cells), in triplicate. The cells were monitored for another 2 days with the RTCA system, and impedance was plotted over time. Cytolysis was calculated as (impedance of target cells without effector cells—impedance of target cells with effector cells) × 100/impedance of target cells without effector cells.

### 4.7. Cytokine ELISA Assay

The target cells were cultured with the effector cells (CAR-T cells or non-transduced T cells) at a 1:1 ratio (1 × 10^4^ cells each) in U-bottom 96-well plates with 200 μL of AIM V-AlbuMAX medium containing 10% FBS, in triplicate. After 16 h the top 150 μL of medium was transferred to V-bottom 96-well plates and centrifuged at 300 g for 5 min to pellet any residual cells. In some experiments supernatant after RTCA assay at E:T=10:1 was used for cytokine ELISA assays. The supernatant was transferred to a new 96-well plate and analyzed by ELISA for human cytokine levels (IFN-gamma, IL-2, IL-6) using kits from Thermo Fisher (South San Francisco, CA, USA) according to the manufacturer’s protocol.

### 4.8. Mouse Xenograft Tumor Growth

Six-week old male NSG mice (Jackson Laboratories, Bar Harbor, ME, USA) were housed and manipulated in strict accordance with the Institutional Animal Care and Use Committee (IACUC). Each mouse was injected subcutaneously with 2 × 10^6^ BxPC3 cells in sterile 1xPBS and then CAR-T cells were injected intratumorally with three doses: 2 × 10^5^ cells/mice; 2 × 10^6^ cells/mice; 2.8 × 10^6^ cells/mice doses at days 20, 27, and 34 respectively, and the xenograft tumor growth was analyzed. Tumor sizes were measured with calipers twice-weekly and tumor volume (in mm^3^) was determined using the formula W^2^L/2, where W is tumor width and L is tumor length. Tumors were excised and fixed in 4% paraformaldehyde, then embedded in paraffin wax and stained by immunohistochemistry. At the end of the intravenous CAR-T cell study, 100 μL of blood was collected and stained with different antibodies by flow cytometry as indicated above.

### 4.9. Immunohistochemistry (IHC) Staining

Tumor tissue sections (4 μm) were incubated in xylenes twice for 10 min, then hydrated in graded alcohols and rinsed in 1× PBS. Antigen retrieval was performed for 20 min in a pressure cooker using 10 mM citrate buffer, pH 6.0. The sections were cooled, rinsed with PBS, incubated in a 3% H_2_O_2_ solution for 10 min, and then rinsed with 1× PBS. The tissue sections were incubated in goat serum for 20 min and then incubated with rabbit anti-cleaved caspase-3 (Asp175, Cell Signaling Technology, Danvers, MA, USA) or rabbit IgG (Jackson Immunoresearch, West Grove, PA, USA) at 0.2 μg/mL overnight at 4 °C. The sections were rinsed with PBS, incubated with biotin-conjugated goat anti-rabbit IgG for 10 min, rinsed with PBS, incubated with streptavidin-conjugated peroxidase for 10 min, and rinsed with PBS. Finally, the sections were incubated in DAB substrate solution for 2–5 min, immersed in tap water, counterstained with hematoxylin, rinsed with water, and dehydrated in graded alcohols and xylenes. Coverslips were mounted with glycerin. Images were acquired on a DMB5-2231PL microscope (Motic, Xiamen, China) with Images Plus 2.0. software.

### 4.10. Humanization of CD47 Antibody

The mouse B6H12 CDR was used for humanization using IgBLAST (NCBI) software according to the methods described [22,23]. The human frames of human clones with the highest homology were used for humanized pairs. Mouse CDR were inserted into these clones and four different variants were generated for testing by ELISA.

### 4.11. Binding Assay with Humanized and Mouse CD47 scFv

The mouse or humanized CD47 ScFv contained VL and VH sequences that were linked with G4Sx3 linker. The ScFvs were fused in frame with C-terminal human Fc inside pYD11 vector used for recombinant CD47 ScFv protein expression. The supernatant with mammalian expressed ScFv protein were used for binding assay at equal amount. All ScFv were checked by Western blotting with anti-human Fc antibody for expression. The human extracellular domain (19–41 amino-acids) of human CD47 protein (Gen Bank ID: NM_001777.3) was fused with mouse Fc and used for ELISA assay with CD47 ScFv. The OD reading at 450 nm was used for detecting binding. The in silico model was generated for humanized VH and VL sequences based on the mouse sequences.

## 5. Statistical Analysis

Data were analyzed and plotted with Prism software (GraphPad, San Diego, CA, USA). Comparisons between two groups were performed by unpaired Student’s t test, and comparisons between three groups were performed by one-way ANOVA with Tukey’s post-hoc test, except where noted. The difference was considered significant with *p*-value < 0.05.

## 6. Conclusions

This is the first report that demonstrates the efficacy of CD47-CAR-T cells against different cancer cells in vitro and in vivo in a pancreatic BcPC3 xenograft model. The use of CD47-CAR-T cells provides a novel type of anti-cancer cellular therapeutics. Future studies will be needed to optimize CD47-CAR-T cell therapy for different types of cancer.
